# Drive‐up INR testing and phone‐based consultations service during COVID‐19 pandemic in a pharmacist‐lead anticoagulation clinic in Qatar: Monitoring, clinical, resource utilization, and patient‐ oriented outcomes

**DOI:** 10.1002/jac5.1469

**Published:** 2021-05-20

**Authors:** Eman N. Alhmoud, Osama Badry Abd El Samad, Hazem Elewa, Ola Alkhozondar, Ezeldin Soaly, Rasha El Anany

**Affiliations:** ^1^ Al Wakra Hospital Hamad Medical Corporation Doha Qatar; ^2^ College of Pharmacy, QU Health Qatar University Doha‐ Qatar

**Keywords:** anticoagulation, COVID‐19, telehealth

## Abstract

**Background:**

Coronavirus disease 2019 (COVID‐19) pandemic has resulted in unprecedented pressure on healthcare systems and led to widespread utilization of telemedicine or telehealth services. Combined with teleclinics, using drive‐up fingerstick International normalized ratio (INR) testing was recommended to decrease exposure risk of anticoagulation patients.

**Objective:**

To evaluate the impact of transitioning from clinic‐based anticoagulation management services to drive‐up and phone‐based services during COVID‐19 pandemic in Qatar.

**Methods:**

The study comprised of two components: a retrospective cohort study of all eligible patients who attended anticoagulation clinic over 1‐year period (6 months before and 6 months after service transition) and a cross‐sectional survey of eligible patients who agreed to provide data about their satisfaction with the new service. Monitoring parameters, clinical outcomes, and resource utilization related to warfarin therapy were compared before and after service transition. Patients' experience was explored through a structured survey.

**Results:**

There was no statistically significant difference between clinic‐based and phone‐based anticoagulation services in mean time and number of visits within therapeutic range (*P* = .67; *P* = .06 respectively); mean number of extreme subtherapeutic and supratherapeutic INR values (*P* = .32 and *P* = .34, respectively); incidence of thromboembolic complications and warfarin related hospitalization. There was one reported bleeding and one emergency visit (0.9%) in the phone‐based group vs none in the clinic‐based group. Frequency of INR testing and compliance to attending clinics appointments declined significantly (*P* = .002; *P* = .001, respectively). Overall, patients were highly satisfied with the new service. The majority of patients found it better (51.6%) or just as good as the traditional service (44.5%). Patients who preferred the new service were significantly younger than their counterparts (*P* = .005).

**Conclusion:**

The service of drive‐up INR testing and phone‐based consultations was shown to be comparable to traditional anticoagulation service, a finding that supports maintaining such services as part of the new normal after the pandemic is over.

## 1 BACKGROUND

The global pandemic of Coronavirus disease 2019 (COVID‐19) has resulted in unprecedented pressure on economics and healthcare systems and created the biggest healthcare crisis in the century.^1^ With more than 2 million reported deaths,^2^ the crisis necessitated public health strategies to reduce the risk of COVID‐19 transmission, preserve personal protective equipment, and accommodate patient surges on facilities while maintaining access to essential health services. Thus, the practice of telemedicine was globally advocated and adopted.^3^, ^4^

Advanced age and concurrent comorbid conditions are well‐recognized predictors of poor COVID‐19 outcomes.^5^, ^6^ A recent study of anticoagulation clinics in Qatar demonstrated that 63% of patients were hypertensive, 59% had diabetes, and 11% had chronic heart failure.^7^ Alongside the necessity for close follow‐up and International Normalized Ratio (INR) monitoring, such patients are at particularly higher risk of COVID‐19 exposure and complications.

Strategies recommended to decrease the risk of COVID‐19 exposure in this population include switching to direct oral anticoagulants (DOAC), transitioning to patient self‐testing, extending INR monitoring intervals, and using drive‐up fingerstick INR testing, which eliminates the need to enter the clinic or facility.^8^ Some of these strategies were adapted by anticoagulation services in Qatar.^9^, ^10^

The practice of phone‐based anticoagulation management service (AMS) was described since the 1990s, particularly for homebound and rural populations.^11^ Multidisciplinary, phone‐based AMS was associated with higher patients' knowledge about warfarin and better satisfaction with care when compared with traditional physician‐based practice.^12^

Moreover, anticoagulation management delivered via telehealth (phone or web‐based consultations) yielded similar clinical and surrogate outcomes in most comparisons to specialized face‐to‐face anticoagulation clinics^13^, ^14^, ^15^ and better outcomes than usual care management.^14^, ^16^ Al‐Wakra hospital's anticoagulation clinic was the first to adapt the service of drive‐up (drive‐through) anticoagulation testing, combined with telehealth consultations, in the Middle East and North Africa (MENA) region.^9^

The aim of this study is to evaluate the impact of transitioning from clinic‐based anticoagulation management services to drive‐up and phone‐based services during COVID‐19 pandemic in Qatar.

## 2 METHODS

### 2.1 Study design and ethics

The study was consisted of two components.

Phase 1: A retrospective cohort study of all eligible patients who attended the anticoagulation clinic at AWH over 1‐year period (6 months before and 6 months after service transition).

Phase 2: A cross‐sectional survey of eligible patients who agreed to provide data about their satisfaction with the new service.

The study was deemed as “service evaluation project,”, thus Institutional Review Board (IRB) review and approval was waived.

### 2.2 Study setting

The study was conducted in Al‐Wakra anticoagulation clinic, one of three specialized anticoagulation clinics in Qatar. The clinic operates 5 days/week and is staffed by one full‐time equivalent (FTE) clinical pharmacy specialist and one FTE nurse. Pharmacists providing anticoagulation services in Qatar must hold a post‐graduate degree in clinical pharmacy, have a minimum 3‐year experience, and complete specialized education and training in anticoagulation management. In April 2020, the time when COVID‐19 cases started to rise in Qatar, the anticoagulation service was shifted from in‐person clinic visits where point‐of‐care (POC) INR was checked and consultations provided to drive‐up INR testing and phone‐based consultations.^9^

Patients were requested to drive‐up a designated lane to the testing spot where the anticoagulation nurse confirmed patients' identity and performed the standard POC INR testing. Results were wirelessly transferred to the patients' electronic medical record (Cerner) and verified by the clinic's pharmacist who subsequently called the patients and conducted a teleconsultation. The consultations were structured similar to those in the clinic where the pharmacist gathered relevant information, decided on dosing regimen and next follow‐up appointment, and reinforced patient education. Patients with INR values above five were instructed to repeat the test via venipuncture in the hematology lab, which is located inside the building before proceeding with the consultation. The service was provided by the same pharmacist and nurse, using the same POC INR testing device throughout the study period.

In contrast to the low‐priced consultation fees that non‐exempted patients pay for face‐to‐face visits, the service was provided free of charge for all patients.

### 2.3 Study population and timeline

For the cohort study (phase 1), retrospective electronic chart review of all consecutive adult patients (≥18‐years old) who received warfarin therapy and visited AWH anticoagulation clinic for a minimum of 6 months before and 6 months after service transition was conducted. Exclusion criteria included pregnancy, hospitalization during the study period, warfarin therapy interruption for >1 week, and less than three retrievable INR measurements in each 6 month‐period before and after service transition.

The patient satisfaction survey (phase II) included all service recipients who visited the clinic before and after transition and agreed to complete the survey between the dates of October 20 and November 20, 2020.

### 2.4 Data collection

Data, including demographic characteristics, indication for warfarin therapy, duration of anticoagulation, target INR range, number of clinic visits, INR at each visit, incident bleeding and/or thromboembolic events, warfarin‐related emergency department (ED) visits, and hospitalizations, were collected by medical chart review through Cerner. INR values were considered therapeutic if they lie within 0.2 units of the target range. Extreme subtherapeutic INR (≤1.5) and extreme supratherapeutic INR (≥4.5) classification was used as previously described by Shulman and colleagues.^17^

Clinical events were defined as thromboembolic events and bleeding events. Thromboembolic events included deep vein thrombosis, pulmonary embolism, systemic embolism, cerebral vascular accident, and/or transient ischemic attack. Bleeding events were classified into major bleeding and/or clinically relevant non‐major bleeding, according to internationally recognized criteria.^18^, ^19^

Patient satisfaction data were collected by staff nurses through interviews using a structured patient survey. The survey was developed based on a thorough literature review of existing patient satisfaction surveys, particularly in the field of telemedicine.^12^, ^20^, ^21^, ^22^, ^23^, ^24^ It was then assessed for face validity by two experts in the field and pilot‐tested for content validity and clarity by three pharmacists and two nurses with feedback incorporated into the final survey.

The survey consisted of four domains.

Domain 1: demographic information, including age, gender, occupation, and educational level. Domain 2: aspects of care, which consisted of 10 questions addressing the three main aspects of care: quality of care (5 questions), access issues (3 questions), and interpersonal issues (1 question), followed by an overall satisfaction assessment (1 question). Respondents rated their agreement on relevant statements on a 5‐element Likert scale where 5 indicated “strongly agree” and 1 indicated “strongly disagree.” Domain 3: Compared the new service to the conventional service. Patients were requested to rate their current experience compared with traditional clinic visits on a four‐point‐scale as: better than a traditional visit; just as good as; worse; or not sure. Additionally, patients were requested to indicate how likely they were to continue using the new service after the COVID‐19 pandemic ends and how likely would they recommend it to someone else on a five‐point scale as: definitely will; probably will; probably will not; definitely will not; not sure. If the answer to the first question was probably will not; definitely will not; not sure, the respondent was asked an open‐ended question to identify the main reason for preferring the traditional service. Domain 4: included two open‐ended questions. “What do you like best about the new service?” and “What can we do to improve?”

To avoid potential bias, neither the clinic's pharmacist nor the nurse was involved in conducting patients' interviews. All survey responses were kept anonymous.

### 2.5 Study outcomes

The objective of the study was to compare the quality of anticoagulation management among patients attending anticoagulation clinic before and after the transition from clinic‐based INR testing and consultations to drive‐up testing and teleconsultations, through (a) anticoagulation quality outcomes (including time in therapeutic range [TTR] as calculated by the standard linear interpolation method described by Rosendaal and colleagues,^25^ frequency of visits with therapeutic INR values, and incidence of extreme sub‐ and supra‐therapeutic INR values [INR less than 1.5 or more than 4.5, respectively]); (b) clinical outcomes (including the incidence of thromboembolic and bleeding complications); (c) resource utilization (including frequency of INR checks, compliance to attending clinic appointments, and warfarin‐related hospitalizations and ED visits), and (d) patients' satisfaction (assessed by a structured survey).

### 2.6 Statistical analysis

Descriptive statistics were used to analyze baseline demographics. Depending on their normal distribution, numerical data were presented as mean ± SD or median and interquartile range (IQR). Continuous variables were tested for normality tests using Kolmogorov‐Smirnov. Categorical variables were presented as frequencies and percentages and analyzed using Chi‐squared test.

Based on the type of data analyzed, pre and post‐service transition outcomes were compared by paired *t* test and McNemar's Chi‐square test.

For the cohort study, a sample size of ~100 subjects was found sufficient to detect TTR difference of 10% with SD of ±15 considering alpha error of 5% and 90% power. The sample size calculator by Raosoft Inc. was used for the survey part.^26^ Utilizing the margin error of 5%, confidence level of 95%, population size of 150 (estimated number of patients following in the clinic) and response distribution of 50%; and non‐response rate of 15%, the minimum required sample size was 125.

Patients' overall satisfaction was rated as high (4, 5 on Likert scale); neutral (3) and low (2, 1).

Adopted from Polinski and colleagues, patient's preference of the new service compared with the traditional service was based on responses to the question “How did your drive‐up and phone visit overall experience compare to a traditional in‐person clinic visit?” Responses were categorized into “patient prefers new service” if the response was “better than a traditional visit” and “patient likes new service” if the response was “better than,” or “just as good as” a traditional visit.^20^

The relationship between patients' demographics and their overall satisfaction and preference of the new service over the traditional one was analyzed by univariate analysis.

A *P*‐value of less than .05 was considered statistically significant. All statistical tests were carried using the IBM Statistical Package for Social Sciences, SPSS (IBM Corp., Armonk, New York) version 26.

## 3 RESULTS

The cohort study included 108 patients while the satisfaction survey was submitted to 129 subjects among which 128 responded (response rate 99.2%). Demographic data were collected for survey respondents (Table 1). The majority of patients were males (67.4%) and were of Middle Eastern (67%) origin. Mean age was 51.2 ± 15.2 years and 43% of patients received warfarin for 1 to 5 years. The most common indication for anticoagulation was atrial fibrillation (31.3%).

**TABLE 1:** Participants demographics (survey part), (n = 128)

Age (mean ± SD)	51.2 ± 15.2 years
Gender N (%)	
Female	41 (32%)
Male	87 (68%)
Nationality N (%)	
Country of origin (according to WHO regional classification)	
Eastern mediterranean	86 (67%)
South‐East Asia	38 (30%)
Europe	2 (2%)
Western Pacific	1 (1%)
Indication of warfarin N (%)	
Atrial fibrillation	40 (31%)
Valve replacement	34 (27%)
Deep vein thrombosis (DVT)	15 (12%)
Pulmonary embolism (PE)	5 (4%)
Combined DVT and PE	5 (4%)
Splanchnic vein thrombosis (splenic, mesenteric, portal veins)	7 (5%)
Left ventricular thrombus	6 (5%)
Cerebral venous thrombosis	3 (2%)
Other indications	13 (10%)
Number of years on warfarin N (%)	
<1 year	20 (16%)
1–5 years	55 (43%)
>5–10 years	30 (23%)
>10 y	23 (18%)
Occupation N (%)	
Working	66 (52%)
Retired	28 (22%)
No current job	34 (27%)
Highest academic degree N (%)	
Below high school level	22 (17%)
High school graduate	43 (34%)
College/university level graduate	63 (49%)

### 3.1 Monitoring parameters

There was no statistically significant difference in mean TTR before and after service transition (82.3 ± 19.4 before vs 83.4 ± 18.4 after; *P* = .67) (Table 2). The percentage of visits with INR values within therapeutic range was comparable between the traditional and new service (68.4% ± 16.9 vs 64.3% ± 16.8, *P* = .06). To eliminate the initiation phase effect (first 6 weeks of therapy), data were reanalyzed after excluding the new warfarin patients (n = 9). Both TTR and percentage of INR values within therapeutic range remained comparable between the two groups (*P* = .27, *P* = .09, respectively).

**TABLE 2:** Comparison of monitoring, clinical, and resource utilization outcomes between traditional and new anticoagulation services

Outcome	Traditional clinic‐based INR testing and consultations	New drive‐up INR testing and phone‐based consultations	*P* value
TTR^a^ (mean ± SD)	82.3 ± 19.4	83.4 ± 18.4	0.67
INR tests within therapeutic range^a^	68.4 ± 16.9	64.3 ± 16.8	0.06
Extreme sub‐therapeutic INR^a^	4.5 ± 9.6	3.4 ± 7.6	0.32
Extreme supra‐therapeutic INR^a^	1.5 ± 3.9	2.1 ± 5.1	0.34
Number of INR tests^a^	9.6 ± 5.6	8 ± 5.3	0.002
Compliance to clinic visits (show‐up)^a^	88.3 ± 11.5	65.8 ± 21.5	<0.001
Thromboembolic events^b^	1 (0.9%)	1 (0.9%)	1
Bleeding outcomes^b^	0 (0%)	1 (0.9%)	^c^
Warfarin‐related hospitalizations^b^	1 (0.9%)	2 (1.8%)	1
Warfarin‐related emergency visits^b^	0 (0%)	1 (0.9%)	^c^

^a^ Expressed as (mean ± SD).

^b^ Expressed as Number (percentage).

^c^ No statistics are computed because Bleeding and warfarin‐related emergency visits (Before) is a constant.

Additionally, there was no difference in mean number of extreme subtherapeutic and supratherapeutic INR values between the two groups (*P* = .32 and *P* = .34, respectively).

### 3.2 Clinical outcomes

No difference in the incidence of thromboembolic complications was noted in the two groups. For bleeding outcomes, only one patient in the phone‐managed group experienced major bleeding (vaginal bleeding that resulted in >2 g/dL drop in hemoglobin) compared with no patients in the clinic‐managed group.

### 3.3 Resource utilization

The frequency of clinic visits and INR testing was significantly lower in the post‐service transition period. Mean number of visits declined from 9.6 ± 5.6 to 8 ± 5.3, *P* = .002. There was also a significant decline in attendance to scheduled clinic appointments from a mean percent of 88.3 ± 11.5% to 65.8 ± 21.5%, *P* < .001. Warfarin‐related hospitalization was comparable between the two groups (*P* = 1). One patient in the phone‐based group (0.9%) visited ED for a warfarin‐related complication compared with none in the clinic‐based group.

### 3.4 Patient satisfaction

Patients' experience with the new service was remarkably positive. Table 3 describes breakdown of patients' responses. Almost all patients (99%) were highly satisfied with the accessibility, quality and interpersonal aspects of the new service. Patients were least satisfied with the quality of communication provided about the service logistics upon its inception (eg, location and process of testing and consultations). Overall, 127 patients (99.2%) were highly satisfied with all aspects of the new service. Only one patient reported neutral response, and none was dissatisfied.

**TABLE 3:** Participants experience with the new drive‐up and phone‐based anticoagulation service (breakdown of responses to domain 2 and 3 questions)

	Domain 2: Aspects of care	N (%)			
	Aspect of care	Statement	Strongly agree/agree	Neutral	Disagree/strongly disagree
1	Quality	The new service (ie, purpose, location and process of testing and consultations) was clearly communicated to you	114 (89)	1 (1)	13 (10)
2	Access	The drive‐up location was practical and accessible	123 (96)	1 (1)	4 (3)
3	Access	The waiting time for drive‐up testing was acceptable	127 (99)	1 (1)	0
4	Access	The clinician called to update you about the INR test result and provided phone consultation in a timely fashion (within 20 minutes)	127 (99)	1 (1)	0
5	Quality	You found it easy to hear and understand instructions provided by the clinician by phone	127 (99)	1 (1)	0
6	Quality	The clinician listened to you carefully and addressed all your concerns/questions	127 (99)	1 (1)	0
7	Quality	The clinician spent enough time with you	127 (99)	1 (1)	0
8	Quality	The treatment plan and education were clearly communicated by the clinician	127 (99)	1 (1)	0
9	Interpersonal	The clinic staff treated you with courtesy and respect	127 (99)	1 (1)	0
10	Overall	You are generally satisfied with the new service	127 (99)	1 (1)	0
	**Domain 3: New versus Conventional service**	**N (%)**			
	**Statement**	Better	Just as good	Worse	Unsure
1	How did your drive‐up and phone visit overall experience compare to a traditional in‐person clinic visit?	66 (52)	57 (44)	5 (4)	0
	**Statement**	**Definitely/probably will**	**Unsure**	**Probably/definitely will not**	
2	How likely would you be to use the drive‐up and phone follow up service at AWH once the COVID‐19 pandemic is over?	113 (88)	0	15 (12)	
3	How likely would you be to recommend drive‐up and phone visit at AL‐Wakra Hospital to someone else?	122 (95)	1 (1)	5 (4)	

When compared with traditional service, almost half of the patients (66, 51.6%) preferred the new service and 57 patients (44.5%) found it “as good as the traditional one”. Only 15 patients (12%) reported that they “probably will not” or “definitely will not” use the new service once the COVID‐19 pandemic is over despite the fact that 11 out of those 15 (73.3%) indicated that it is “as good as the traditional one.” Eleven patients responded to the question “what is the main reason that makes you prefer clinic‐based testing and consultations” by saying that they prefer direct contact with the clinician, of those five patients added that they prefer having their vital signs checked each visit, which was not the case with drive‐up testing. In response to the open‐ended question “what do you like best about the new service?” timeliness, convenience, and limiting COVID‐19 exposure risk were the most common answers. With regard to the open‐ended question “What can we do to improve?” the most responses described the service as an innovative and well‐organized service that does not require any further improvements. Few patients suggested adding vital signs measurement at the INR testing spot.

None of the demographic variables evaluated predicted patients' overall satisfaction, preference, or likeness for the service except age. Patients who preferred the current service over the traditional one were significantly younger than those who found it either as good or worse (47.6 ± 15 vs 55 ± 14.5, *P* = .005).

## 4 DISCUSSION

The current study explored the clinical efficacy and safety as well as resource utilization and patient satisfaction accompanying the transition of traditional clinic‐based AMS to drive‐up testing and teleconsultations in response to COVID‐19 pandemic. It revealed that neither monitoring nor clinical outcomes related to warfarin therapy were significantly impacted by service transition. Apart from an expected decline in number of clinic visits, the new service did not yield any increases in warfarin‐related hospitalizations or ED visits. Furthermore, patients' satisfaction with the new service was impressive.

The study findings of comparable outcomes between traditional clinic‐based AMS and phone‐based ones are in line with findings of some previous studies while they contradict with others. Wittkowsky and colleagues^13^ were the first to compare outcomes of phone vs clinic‐based AMS. Among 234 patients, the two modalities were comparable in terms of number of therapeutic INR values, frequency of clinic visits, and frequency of hemorrhagic and thromboembolic events as well as warfarin‐related emergency and hospital visits. However, phone consultations were provided to patients if they could not afford clinic visits due to health or social issues, and variations in staffing levels and practitioners providing care were not excluded. Findings that may have influenced compliance to treatment and clinical outcomes. In contrast, the current service was provided to all patients by the same practitioner over the course of the study.

A later study by Cryder and colleagues confirmed the equivalence of traditional and telephonic AMS in terms of both surrogate and clinical outcomes, and their superiority over usual physician‐based care.^14^

On the other hand, in a study of 110 patients, phone‐based encounters were associated with more INR checks per patient‐year; a lower percentage of therapeutic INR values; and a 2‐fold increased incidence of extreme out‐of‐range INR values compared with office visits. Nonetheless, overall TTR was similar between the two groups.^15^ Similar to the work of Wittkowsky and colleagues^13^ and contrary to this study, telephonic service was limited to nonambulatory and homebound patients as well as those living at distant locations. Thus, increased fluctuations in therapy could have potentially resulted from complicated disease states rather than consultation modality. The study authors hypothesized that the lag time in addressing INR values could have contributed to the finding of more frequent subtherapeutic INR values. Delays in communicating INR results were not addressed by Wittkowsky and colleagues and were not applicable in the current study.

Compared with traditional physician‐based practice, a larger study by Witt and colleagues (6645 patients), revealed that patients managed by specialized, pharmacist driven, phone‐based AMS spent significantly longer period within the target INR range, had a lower percentage of extreme INR values, and were 39% less likely to experience any anticoagulation therapy‐related complication.

In contrast to our service, were phone visits replicated the model of in‐person visits, better outcomes in the previous study could be confounded by discrepancies in expertise of practitioners providing care and monitoring systems utilized (computerized vs basic paper‐based systems).^16^

Superior outcomes associated with a virtual warfarin management model utilizing specialized telehealth audio/visual software were also revealed in a recent small study from New York.^27^

The number of clinical events reported in the current study is low. This could be attributed to the small sample size and relatively short follow‐up period. Furthermore, these findings are parallel to results of previous studies indicating low warfarin related complications in Qatar's population.^28^, ^29^ With regard to resource utilization after service transition, the current study revealed a significant decline in the frequency of INR testing and patients' compliance to attending scheduled clinic visits (a mean difference of 22.4). This contrasts with a recent study by Zobeck and colleagues, where monthly average patient visits remained steady after implementing a curbside drive‐up anticoagulation clinic when compared with pre‐COVID period.^30^

Findings in this study can be attributed to extending INR monitoring interval for stable patients beyond the 6 to 8 weeks interval recommended by the local protocol along with the national measures placed to ensure social distancing and patients' fear from exposure risk in healthcare facilities. The decline in in‐person visits during COVID‐19 pandemic is evident across other healthcare settings. A cross‐sectional analysis of the United States IQVIA National Disease and Therapeutic Index data, a national audit of outpatient practice, revealed a remarkable decrease in office‐based primary care visits (50.2%), in‐visit monitoring of blood pressure (50.1%) and absolute number of cholesterol assessments (36.9%) during the second quarter of 2020 compared with the second quarters of 2018 and 2019.^30^, ^31^

Another aspect evaluated by this study was patients' satisfaction with drive‐up testing and telehealth anticoagulation management. The provision of telehealth is generally limited by patients' access to hi‐tech devices required to conduct a visit; internet access; level of comfort with technology and cultural acceptance of replacing in‐person visits by virtual ones. Moreover, telehealth had been criticized for threatening the rapport between healthcare providers and patients^32^ and ultimately compromising patients' satisfaction with care. However, available evidence suggests high patient satisfaction with telehealth services, particularly when provided to patients in rural and remote areas.^18^, ^33^

The study findings revealed a high patient satisfaction with all aspects of the service (access, quality, and interpersonal). Most of the patient's either preferred the service or just found it as good as the conventional one.

Waterman and colleagues, was the first to evaluate satisfaction with phone‐based anticoagulation service compared with traditional management by primary‐care physicians. Telephonic AMS was associated with significantly higher patients' and physicians' satisfaction with the service's quality and timeliness and resulted in better patients' knowledge about their anticoagulants.^12^

In the era of COVID‐19, a recent study by Zobeck and colleagues^30^ described the implementation of a drive‐up curbside clinics along with simultaneous in‐person visits, where pharmacists performed POC‐INR testing and provided phone consultations. About half of the respondents (46.6%) preferred drive‐up testing over face‐to‐face visits while 26.7% indicated a preference for the later. Furthermore, while 30.6% of respondents were more likely to continue routine INR monitoring via drive‐up testing than with face‐to‐face visits, about 44% did not believe that the service impacted their likelihood of testing. The authors contributed the findings to the rural nature of the population and the relatively low number of COVID‐19 cases in their area during the survey period, which contrasts with the settings of the current study.

This study has several strengths. Firstly, up to our knowledge, this is the first study that evaluates drive‐up testing of INR and phone‐based anticoagulation consultations in the MENA region.

Second, concerns about safety, efficacy, resource utilization, and patients' satisfaction were all addressed. Additionally, the service was provided by the same practitioner throughout the course of the study to all patients visiting the clinic which eliminated potential time and selection bias reported in previous literature.^13^, ^27^

Moreover, the simplicity of the phone follow‐up service without requiring extra resources to operate and maintain such as devices with cameras and internet access made it easier to adapt and sustain.

The study findings, nonetheless, may not be generalizable to healthcare institutions or clinics with different patient populations or healthcare delivery models, or those that switched to virtual visits using video meeting technology instead of telephone only.

In conclusion, the current study confirms that the new drive‐up INR testing and anticoagulation teleconsultations provide optimal anticoagulation quality while maintaining acceptable resource utilization and patient satisfaction. A Finding that suggests integrating such service to traditional care delivery even after the pandemic ends.

## CONFLICT OF INTEREST

The authors declare no conflicts of interest
